# A Novel Mutation of CADHERIN (*CDH15*) in an Iranian Boy With Borderline Intelligence Without Dysmorphism—A Case Report

**DOI:** 10.1002/ccr3.71511

**Published:** 2025-12-07

**Authors:** Mahmoud Reza Ashrafi, Ali Nikkhah, Morteza Heidari, Solmaz Aziz‐Ahari, Zahra Rezaei, Ali Rashidi‐Nezhad, Golazin Shahbodagh Khan, Roya Sinaei, Shiva Bayat

**Affiliations:** ^1^ Pediatrics Center of Excellence, Department of Pediatric Neurology, Children's Medical Center Tehran University of Medical Sciences Tehran Iran; ^2^ Epilepsy Monitoring Unit, Pediatric Neurology Ward, Children's Medical Center of Excellence Tehran University of Medical Sciences Tehran Iran; ^3^ Maternal, Fetal and Neonatal Research Center, Family Health Research Institute, Vali‐Asr Hospital, Imam Khomeini Hospital Complex Tehran University of Medical Sciences Tehran Iran; ^4^ Department of Medical Genetics, School of Medicine Tehran University of Medical Sciences Tehran Iran

**Keywords:** *CDH15*, intellectual disability, intrafamilial variability, penetrance

## Abstract

This case study presents a case of genetic intellectual disability that has been transmitted from a seemingly normal mother to her son, which exemplifies the occurrence of reduced penetrance of the condition, a mechanism already known to be implicated in the transmission of autosomal dominant neurological conditions.

## 1 Introduction

Different genetic mechanisms are known to be implicated in the etiology of neurodevelopmental and neuropsychiatric disorders, be it in the single gene or the complex form, which result from the combinatorial effect of many moderate‐effect variants. One of the aforementioned mechanisms is reduced penetrance in genes implicated in autosomal dominant neurological conditions, rendering them transmissible from one generation to the next. This phenomenon can create a range of clinical presentations from no phenotypic manifestations in one end of the spectrum to severe neurodevelopmental presentations in the other end, which can happen with intrafamilial and interfamilial variation, leading to the missing diagnosis of some individuals with very mild features, sometimes overlapping those in the general population, which is the case in this report.

This case report is one of the many reported cases by far, dissecting the role of reduced penetrance in an intellectual developmental disorder noticed in the son and to our surprise, traced back to the mother. The patient is a 7‐year‐old boy with developmental delay in gross motor and verbal milestones who was subsequently referred for genetic testing after complete laboratory and metabolic workup in addition to comprehensive neurological evaluation, all of which remained ambiguous about the underlying condition of the child. It presents a novel frameshift mutation with no previous reports in *CDH15*, the gene encoding Cadherin 15, a transmembrane glycoprotein implicated in cellular adhesion.

*CDH15*, encodes a calcium‐dependent cellular adhesive belonging to the Cadherin superfamily, proteins well known for their pivotal role in brain development, as well as maintaining synaptic structure, function, and plasticity, altogether implicating them in the etiology of genetic intellectual disability. Heterozygous variants in *CDH15* have been previously reported as factors implicated in the etiology of autosomal dominant intellectual disability type 3.

Therefore, this case report pinpoints the significance of genetic testing in cases of intellectual disability with unknown etiology, specifically in cases of inconclusive metabolic and neurological workup. It additionally intensifies the occurrence of reduced penetrance and the possibility of transmission of variants from seemingly normal parents, who may be identified as mild ID through further evaluations.

## 2 Case History/Examination

The patient is a 7‐year‐old boy with developmental delay in gross motor and verbal milestones. The older sibling is a 14‐year‐old girl with no abnormalities regarding her development and no dysmorphism. He is the second child from consanguineous parents with no history of delivery complications. He is able to walk unaided and make simple sentences at age 2 following occupational therapy and speech therapy. The father is a 40‐year‐old man, with sixth‐grade education, self‐employment and no dysmorphism. The proband's mother, a 34‐year‐old woman with no facial dysmorphism is fully capable of performing her daily activities.

He currently presents with wide‐based and ataxic gait and speech disfluency with no facial dysmorphic features and no history of seizures. Additionally, he displays behavioral abnormalities including hyperactivity, talkativeness and lack of attention in favor of ADHD.

## 3 Differential Diagnosis, Investigations and Treatment

No specific differential diagnoses were indicated for the patient. Wechsler Intelligence Scale for Children test (WISC_IV) was applied for ranking the proband in terms of verbal comprehension, working memory, perceptual reasoning and processing speed. Comprehensive Laboratory tests encompassing complete blood count, thyroid and liver function test, serum immunoglobulins, serum amino acids, alpha fetoprotein and creatine phosphokinase (CPK) were also requested for further evaluation of the patient. Furthermore, EMG‐NCV and brain MRI were performed.

To investigate the patient' mother, and considering the fact that she terminated her education at elementary school, she also underwent Wechsler Adult Intelligence Scale (WAIS), despite complete ability to fulfill daily activities.

As for genetic testing, after obtaining informed consent from the patient's parents as his legal guardians, DNA was extracted from the patient's peripheral blood utilizing a modified salting out method, which involves lysis of RBCs using RBC Lysis Buffer, lysis of WBCs using ROS, addition of protein precipitation buffe and ultimately washing and rehydration of the precipitated DNA. This process was further followed by target enrichment using the Agilent SureSelect Human All Exon V7 Kits (Agilent Technologies Inc., Santa Clara, CA, USA). Subsequently, the sample was subjected to high‐throughput sequencing on an Illumina NovaSeq 6000 platform (Illumine Inc., San Diego, CA, USA). In‐house filtering pipeline was used for data analysis.

Variants classed as “Pathogenic” according to ClinVar submissions were retained while duplicates, those with a minor allele frequency of > 0.05 in population databases in addition to those located in introns, upstream/downstream regions, intergenic and synonymous variants were filtered out. Consequently, the remaining variants were prioritized according to the functional consequence of variants, frequency in population databases in addition to prediction of variant pathogenicity using in silico tools. Conventional Sanger sequencing was applied to DNA samples of the patient and his parents to confirm the potentially pathogenic variant obtained after data filtering using an ABI 3500 Genetic Analyzer (Applied Bio systems, Foster City, CA, USA).

## 4 Conclusion and Results

**FIGURE 1:**
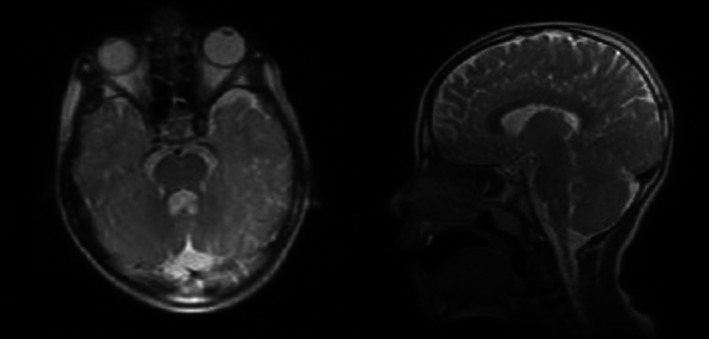
Axial and Sagittal T2 MR Images show inferior vermis hypoplasia and elongation of superior cerebellar peduncle.

With regards to WISC_IV test, the proband received the scores 97,70,72 and 78 in verbal comprehension, working memory, perceptual reasoning and processing speed, respectively with total score 76 classed as “Borderline Intellectual Quotient (IQ).” His mother was also found to possess borderline IQ score 72. The laboratory tests and EMG‐NCV revealed normal results. Brain MRI revealed presence of inferior vermis hypoplasia and elongation of superior cerebellar peduncle as presented in Figure 1.

WES revealed the presence of a novel heterozygous frameshift variant (c.2142_2145del, p.Phe714LeufsTer35) in *CDH15* gene (NM_004933.3). The heterozygosity of the variant was subsequently confirmed by Sanger sequencing in the patient and his mother, with his father displaying homozygosity for the wild‐type variant. Therefore, the variant has been maternally transmitted. The shift in the reading frame leads to premature termination of CDH15 protein and creates a termination codon 35 positions downstream. It is worthy to note that the variant has not been reported in publicly available population databases. Furthermore, the variant has not received any predictions based on in silico tools. The gene is majorly expressed in brain cerebellar hemispheres followed by skeletal muscles.

The patient was advised to undergo occupational therapy on a regular interval and was followed up every 3 months to investigate his progress. No significant progress was made at the time of the study.

## 5 Discussion

Growing evidence has shed light on the fact that proper synaptic formation and plasticity are major prerequisites for normal intellectual function [1, 2]. Cadherin and immunoglobulin superfamily are already known for their pivotal role in brain development, as well as maintaining synaptic structure, function, and plasticity [3]. *CDH15*, a member of the aforementioned Cadherin family and located in the chromosomal position 16q24.3, is the gene encoding the 814‐aa residue Cadherin15, a transmembrane adhesive glycoprotein, known to be implicated in a form of autosomal dominant intellectual disability, namely Intellectual Developmental Disorder, Type 3 (OMIM:612580). It is recognized to be predominantly expressed in skeletal muscles and human brain [4]. Body of evidence altogether suggest that it may be involved in the etiology of ID, either alone or in combination with other contributing factors [5]. Additionally, its dysregulation is pivotal in neuropsychiatric disorders including intellectual disability, schizophrenia and epilepsy [6]. Their role has also been extensively reviewed in major psychiatric disorders [7].

In the case reported by Palumbo et al. [5], the case of a boy with mild‐to‐moderate ID, speech delay and mild dysmorphic features was interrogated and a de novo 16q24.2q24.3 microduplication was found encompassing 9 OMIM genes one of which is *CDH15*. *CDH15* was found to be a prominent contributing etiological factor to the patients' neurodevelopmental manifestations, shared by previously reported patients with overlapping microduplication and phenotypic characteristics [5].

The disruption of the very same gene as a result of t(11;16) in a female patient with ID intensifies the implication of *CDH15* in ID phenotype, reported by Bhalla et al. [8]. The balanced translocation resulted in the disturbance of two cell‐adhesion molecules, CDH15 and KIRREL3, the finding that prompted more search for such variants in a large cohort of unrelated ID cases of unknown etiology which resulted in the detection of *CDH15* non‐synonymous heterozygous variants in functionally constrained regions of the gene in females with mild‐to‐moderate ID. One case of mother‐to‐son transmission of *CDH15* variant was observed in the cohort of patients, both of whom displayed learning and memory problems. The study remained ambiguous about how the two disrupted genes interact to ultimately lead to cognitive deficits warranting the need for further analysis of the variants in other patients.

Reduced penetrance has also been observed in the case of a boy with 16q22.1 microdeletion encompassing *ZFP90* and other members of Cadherin superfamily including *CDH1* and *CDH3*, the mutations which were seemingly maternally transmitted without any phenotypic features in the mother. However, the boy's intellectual disability was attributed to the mutation in *ZFP90* [9].

Twelve cases with a 16q24.2q24.3 were gathered for comparison of clinical and genetic features in the study conducted by Novara et al., all of whom exhibited neurological phenotypes and the deleted segment in the cases included *ANKRD11*, in addition to *CDH15*, *ZFPM1* and *ZNF778* in eight cases. The putative role of *CDH15* in neurological manifestations was further intensified due to the fact that the five patients presenting with cerebral findings in the study all harbored microdeletions involving the *CDH15* gene. It was concluded that haploinsufficiency of *CDH15* culminates in a more severe neurological phenotype with specific regard to brain malformation [10].

In the study on two sibs with neurodevelopmental disorders, two different genetic disorders were identified by CMA and WGS in the younger sister and older brother, respectively. *CDH15* variant was among the variants found in the older brother with moderate ID and ASD in addition to schizophrenia. *CDH15* variant and the other variants were transmitted from unaffected parents, putting forward these variants as moderate‐ effect variants and showing that the older brother's phenotype may be put down to the combination of multiple moderate effect variants, as has already been the case about the genetic mechanisms leading to neurodevelopmental disorders [11].

Although literature exists on the contribution of different genes to the etiology of autosomal recessive intellectual disability in Iran by Najmabadi et al. in 2019, there is paucity of evidence regarding the genes implicated in the autosomal dominant forms, which can be possibly attributed to the challenges posed by incomplete penetrance, variable expressivity and underdiagnosis of cases with presentations at the very mild end of the spectrum, suggesting that the study of autosomal dominant forms of ID can also be prioritized for further studies in the region, ultimately helping to identify new genes, variants and modifier factors contributing to the outcome of such patients [12].

The mechanism of parental transmission in cases of mild ID has also been observed in previously reported cases including the case of loss of 12p11.22 that occurred in two sibs with Borderline Intellectual Functioning (BIF), which had been unwittingly passed on from a seemingly normal father who was also found to harbor the deletion and present with BIF. The results of this study once again pinpoint the significance of genetic studies in cases with intellectual disability in addition to parental analysis taking into account the possibility of transmission of the disease‐causing variant from “normal” parents [13].

CTNNB1, a protein known to be implicated in the formation of cadherin/catenin multiprotein complex, has additionally been reported to underlie severe intellectual disability in the case of a Chinese woman, interestingly having inherited the variant from her mother with slighter clinical presentations, highlighting the role of Cadherin‐associated proteins besides Cadherins in neurodevelopmental processes [14].

Interestingly, in another comprehensive study on genetics of ID in consanguineous families, several genes with an already known autosomal dominant mode of inheritance, were found to be implicated in the recessive forms co‐segregating in consanguineous families, which adds another layer to the complexity of investigation of the molecular underpinnings of ID [15]. The variants reported in previous literature to date have also been summarized and presented in Table 1.

**TABLE 1:** Summary of reports of CDH15 variants in cases of intellectual disability published to date.

Study	Number of patients	Variant ID	Patients phenotype	Year published	Comment
Bhalla et al. [8]	4	p.V8L, p.R60C*CDH15*, p.R92W p.A122V	Mild to severe ID (in all cases)	2008	p.R92W in a female patient with mild ID who transmitted the variant to her son, both had memory and learning deficits
Bayard de Volo et al. [9]	1	A germline 16q22.1 microdeletion encompassing ZFP90, CDH3 and CDH1	Psychomotor delay and dysmorphic features	2012	The deletion was inherited from the mother with no apparent phenotypic features
Novara et al. [10]	12	16q24.2q24.3 deletion	Neurological impairment	2017	
Palumbo et al. [5]	1	16q24.2q24.3 microduplication	Intellectual disability, speech delay and mild dysmorphic features	2020	
Huang et al. [11]	2 sibs (CDH15 variant in one)	p.Asp98Asn	Intellectual disability, autism spectrum disorder, and psychosis	2022	Paternal transmission of the variant from a healthy father

Altogether, the body of evidence put forward *CDH15* as a clinically significant gene implicated in cognitive performance with variable manifestations even between members of the same family. Therefore, more attention must be allotted to diseases with autosomal dominant mode of inheritance that are transferred from one generation to another, putting forward the idea that the variant may be really responsible for the disease but might have been variably presented in the previous generation, that is, reduced penetrance of the variant helped its transmission. Consequently, this case study intensifies the importance of considering disease penetrance in genetic counseling process for better and more accurate interpretation of genomic test results.

## Author Contributions

**Mahmoud Reza Ashrafi:** conceptualization, data curation. **Ali Nikkhah:** investigation. **Morteza Heidari:** formal analysis. **Solmaz Aziz‐Ahari:** investigation. **Zahra Rezaei:** methodology. **Ali Rashidi‐Nezhad:** methodology. **Golazin Shahbodagh Khan:** formal analysis. **Roya Sinaei:** visualization. **Shiva Bayat:** conceptualization, data curation, methodology, writing – original draft.

## Funding

The authors have nothing to report.

## Ethics Statement

The written informed consent was obtained from the parents as legal guardians of the proband. The study protocol was approved by the local medical ethics committee of Children's Medical Center, Tehran, Iran. The study was being performed in accordance with the ethical standards laid down in the 1964 Declaration of Helsinki and its later amendments. The Code of Ethics applicable to this study is IR.TUMS.CHMC.REC.1403.014.

## Consent

The authors affirm that human research participants provided informed consent for publication of the images in Figure 1.

## Conflicts of Interest

The authors declare no conflicts of interest.

## Data Availability

We state that the data not provided in this article will be shared on request by email to the corresponding author from any qualified investigator for purposes of replicating procedures and results.
